# Successful Surgical Management of Urethral Prolapse in a Postmenopausal Female

**DOI:** 10.7759/cureus.38818

**Published:** 2023-05-10

**Authors:** Kevin D Healey, Davong D Phrathep, Andrew B Herson, Kaila R Fives, Jenna R Hurley, Carlos E Ramos, Ahmad O Rifai

**Affiliations:** 1 Urology, Lake Erie College of Osteopathic Medicine, Bradenton, USA; 2 Urology, Advanced Urology Institute, Panama City, USA; 3 Nephrology, The Virtual Nephrologist, Lynn Haven, USA

**Keywords:** lower urinary tract symptoms, luts, urology surgery, urethral prolapse, female urethra

## Abstract

Urethral prolapse is a rare and benign condition where the inner urethral lining protrudes through the external urethral opening. This condition is mostly seen in prepubertal and postmenopausal women. Risk factors include obesity, multiparity, and the onset of menopause. It has a low occurrence, resulting in frequent underdiagnosis. This is compounded by its typical delayed diagnosis. We present a case of a 71-year-old postmenopausal woman who presented with persistent urinary symptoms. After multiple failed conservative treatments, she underwent a successful urethral prolapse excision. Our case highlights the importance of considering urethral prolapse as a differential diagnosis in a postmenopausal woman with continual urinary symptoms.

## Introduction

Urethral prolapse is a condition involving a circular and complete eversion of the distal urethra through the external urethral meatus [1]. It is a rare yet benign condition that predominantly occurs in prepubertal, African American females [2]. Although the etiology of urethral prolapse remains unclear, it is thought to be caused by insufficient estrogen levels or poor attachment between urethral smooth muscle layers [2-3]. In postmenopausal cases, vaginal bleeding is the most common presenting symptom [4]. Other symptoms of urethral prolapse include nocturia, dysuria, hematuria, urinary urgency, and urinary frequency [1]. These symptoms will always be confused as urinary tract infection since urethral prolapse is rare. Treatment options for urethral prolapse include topical antibiotics, sitz baths, vaginal and urethral estrogen creams, and surgical intervention [5]. There is no clear consensus on first-line treatment for urethral prolapse [1]. Our report describes a successful surgical intervention, involving urethral prolapse excision, in the setting of failed conservative measures in a postmenopausal woman with persistent urinary symptoms.

## Case presentation

A 71-year-old woman sought consultation at an outpatient urology clinic, reporting symptoms of dysuria, increased frequency, and urgency of urination for a six-month period. Our patient denies any complaints of incontinence, nocturia, and hematuria. She was seen by her primary care provider who treated her with antibiotics after a negative urine culture, there was no improvement in her symptoms. The abdominal physical examination revealed no abnormalities. A urine dipstick test was negative, but upon microscopic examination, there was a high presence of bacteria (3+). A polymerase chain reaction (PCR) and standard urine culture were obtained, with both being negative.

One week later the patient returned with continued complaints of frequency, urgency, and dysuria. Her frequency had increased in severity. She was recommended to undergo a cystoscopy. One month later, her cystoscopy revealed a normal urethra without stenosis, a normal bladder with minimal trabeculation, and no evidence of urinary retention.

Following the procedure, a trial of Mirabegron 50 mg for one week, followed by tamsulosin for a second week, and then a combination of both for the third week was initiated. 

Upon her return, she reported adverse effects to both medications, and both were subsequently discontinued. Currently, she experiences hematuria and stress incontinence, necessitating the use of multiple pads on a daily basis. A physical exam was then performed which revealed a severely edematous and erythematous urethral meatus. A urine culture was performed and she was treated empirically with Macrobid. She was started on topical estrogen to apply once nightly to the affected area and was advised to trial sitz baths and steroid cream. 

The urine culture was notable for the presence of Escherichia coli and treatment was initiated with the antibiotic Levofloxacin. As, conservative measures had failed in providing relief for her symptoms, the patient decided to proceed with excision of the urethral prolapse.

**Figure 1:**
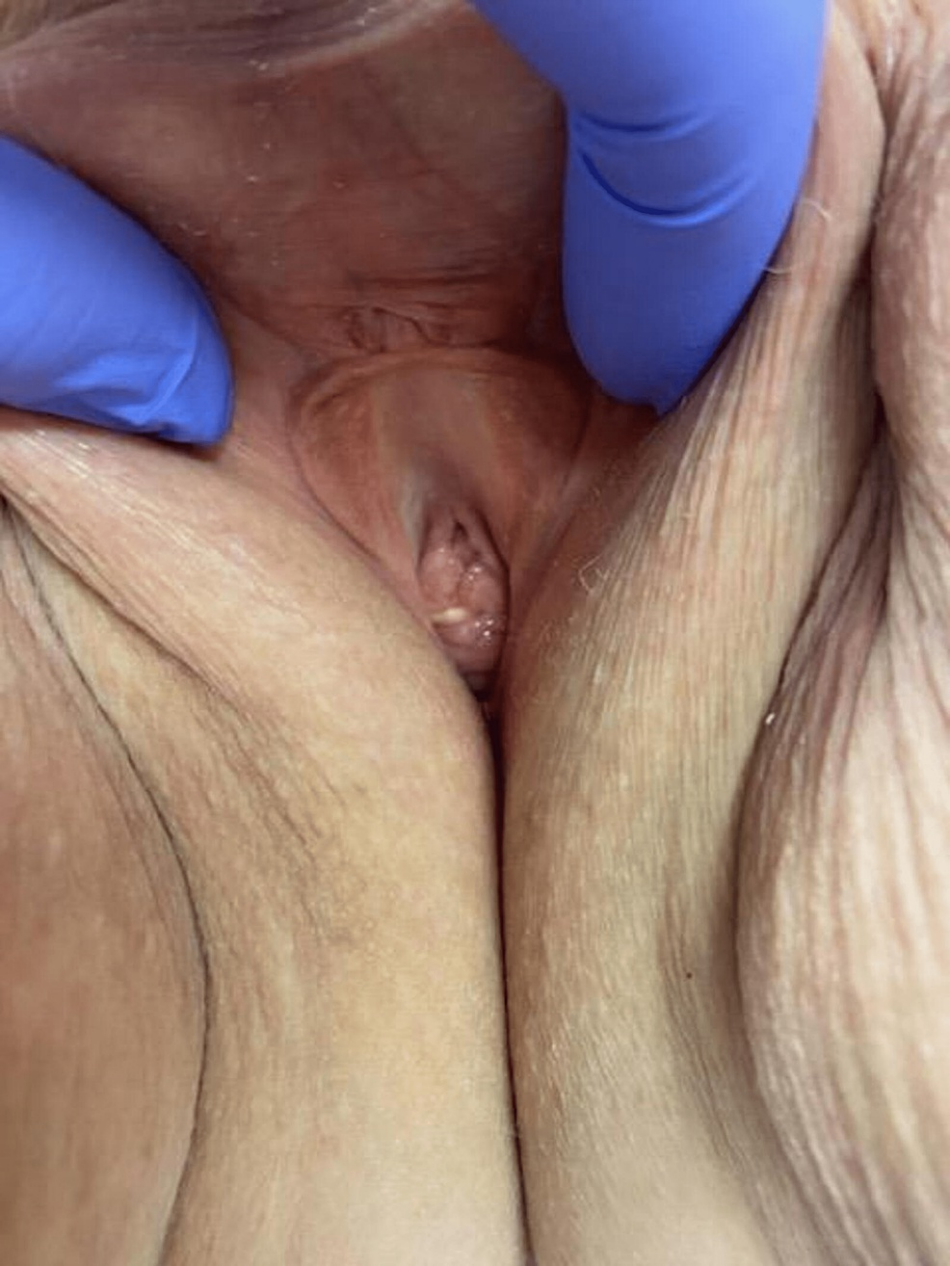
Resolution of prolapsed urethra following surgical excision.

Upon introduction of anesthesia, the patient was placed in the dorsal lithotomy position and the genital area was prepped and draped in standard surgical fashion. The cystoscope was inserted through the urethra and introduced into the urinary bladder which was inspected systematically. The cystoscope was then retrieved and an 18-French Foley was placed and the balloon was inflated with a 10 cc syringe. The everted mucosa was excised and stay sutures were placed at 12 and six o’clock positions. Another set of stay sutures was placed at the three and nine o’clock positions. The intervening spaces were closed with 4.0 chromic in an interrupted fashion. After completion of the surgical procedure, bacitracin ointment was applied to the urethral meatus. The post-operative visit revealed that the healing process had progressed normally and the patient's lower urinary tract symptoms had completely resolved (Figure 1).

## Discussion

Urethral prolapse is a rare condition in which the urethra protrudes through the vaginal opening [2]. Risk factors for urethral prolapse and pelvic floor dysfunction include multiparity, obesity, and menopause [6-7]. The pathophysiology behind urethral prolapse is not well understood, but the main hypothesis stems from the loss of estrogen [8]. The decreased levels of estrogen in postmenopausal women can lead to the development of risk factors such as impaired attachments of the smooth muscle layers around the urethra [1, 8].

 It is imperative that primary care providers recognize the possibility of urethral prolapse as a cause of persistent lower urinary tract symptoms following a course of antibiotics. In patients with mild symptomatic urethral prolapse, there are many treatment options available with variable success. Non-pharmacologic options including lifestyle modifications such as increased physical activity, pelvic floor exercises (e.g., Kegel exercises), sitz baths, and weight loss have been shown to have some efficacy in the treatment of urethral prolapse [9]. Prior literature has also shown pharmacologic options such as topical meatal estrogen, Mirabegron, and topical meatal antibiotics to have some success in the management of symptoms as well [5].

In our case, we report a symptomatic postmenopausal patient who failed prior conservative treatment options such as sitz baths, Mirabegron, tamsulosin, topical meatal estrogen, and topical meatal antibiotics. Although these treatment options show success for some patients, they seem to be more effective in prepubertal females than in postmenopausal women [8]. After conservative measures fail, surgical excision and repair are recommended to relieve symptoms. Many studies have shown a high success rate in these surgeries; stating a lower recurrence rate, higher cure rate, and rapid relief of symptoms in comparison with conservative medical management [8, 10]. This illustrates why surgical excision and repair is the optimal treatment option for postmenopausal women experiencing symptoms that have not been effectively managed through conservative measures.

## Conclusions

Many postmenopausal women present to primary care providers with symptoms such as dysuria, hematuria, nocturia, and increased urinary frequency. Due to its rarity, urethral prolapse often has a delayed diagnosis and is consequently underdiagnosed. Upon physical exam and diagnosis of such conditions, conservative medical management should be promptly initiated. If no improvement in symptoms, surgery should be considered for symptomatic relief, especially in postmenopausal women. With adequate surgical treatment, most postmenopausal women are able to alleviate their symptoms and improve their quality of life. Overall, surgical outcomes in postmenopausal women who suffer from urethral prolapse are generally good with relief of symptoms and a decrease in recurrence rate. However, as with any surgery, there are risks and potential complications involved in any surgical procedure, and women who choose to undergo these procedures should consult the benefits and complications with their respective surgeon and primary care provider.
